# Ultrasound-Guided Radial Artery Compression to Assess Blood Pressure

**DOI:** 10.5811/westjem.2016.12.32344

**Published:** 2017-02-27

**Authors:** Leonard Bunting, Andrew Butki, Ashley Sullivan

**Affiliations:** St. John Hospital and Medical Center, Department of Emergency Medicine, Detroit, Michigan

## Abstract

**Introduction:**

We proposed using compression sonography to observe the coaptation and collapse of the radial artery as a surrogate for automated cuff blood pressures (BP). We hypothesize that the pressure required to achieve coaptation and complete collapse of the artery would correlate to the diastolic and systolic BP, respectively. This pilot study was to assess the feasibility of ultrasound-guided radial artery compression (URAC) for BP measurement and compare patient comfort levels during automated cuff with URAC measurements.

**Methods:**

This was a prospective cohort pilot study with a convenience sampling of 25 adult patients at a single urban emergency department. URAC pressure was measured, followed by cuff manometry on the same arm. A 100mL normal saline bag was connected to the Stryker pressure monitor and placed on the volar wrist. Pressure was applied to the bag with a linear transducer and the radial artery was observed for coaptation of the anterior and posterior walls and complete collapse. Pressures required for coaptation and collapse were recorded from the Stryker display. Patient level of comfort was also documented during the URAC method, with patients reporting either “more,” “same” or “less” comfort in comparison to automated cuffs. We analyzed data using intraclass correlation and paired t-tests. Interrater reliability was calculated using intraclass correlation.

**Results:**

The mean cuff systolic BP was 138.6 ± 22.1 mmHg compared to 126.9 ± 19.8 mmHg for the URAC systolic BP (p=0.02). For diastolic BP, there was no significant difference between the cuff BP and the URAC BP (83.7 ± 13.0 cuff vs. 86.5 ± 19.8 URAC, p=0.46). The intraclass correlation (ICC) for systolic BP was 0.48 (p=0.04) and 0.57 (p=0.02) for diastolic BP. The agreement between the two observers was 0.88 for identifying coaptation on ultrasound (diastolic pressure) and was 0.92 for identifying collapse (systolic pressure). Eighty percent (20/25) of subjects found the URAC method more comfortable than the cuff measurement, and the remainder found it the same (5/20).

**Conclusion:**

This pilot study showed statistically significant moderate correlation between automated cuff diastolic BP and URAC measurements for vessel coaptation. Additionally, most patients found the URAC method more comfortable than traditional cuff measurements. Compression ultrasonography shows promise as an alternative method of BP measurement, though future studies are needed.

## INTRODUCTION

Automated cuff devices are the standard method of measuring blood pressure (BP) in the emergency department (ED). When these devices fail to obtain accurate pressures,^1^ clinicians may resort to invasive methods of determining BP, which have potential complications.^2^,^3^ An alternative noninvasive method of measuring BP using materials readily available in the ED would be helpful.

We propose a new technique to assess intraluminal radial artery pressure using ultrasound-guided radial artery compression (URAC) sonography. It was previously shown that compression ultrasound of the arm can be used by non-vascular sonographers. Thalhammer et. al. successfully measured peripheral venous pressure using compression ultrasound of the cephalic vein and a specialized pressure tranducer.^4^ It has additionally been shown that clinicians can perform compression ultrasound after limited training^5^ and consistently identify the radial artery under ultrasound.^6^

Our technique is novel in that it uses compression sonography to assess arterial pressures and only uses equipment commonly in place in the ED. We hypothesize that the pressure required to achieve coaptation and then collapse of the radial artery on ultrasound will correlate to the standard automated cuff measurements for diastolic and systolic BP, respectively.

## METHODS

Inclusion criteria were any adult patients with a triage automated-cuff pressure reading. Exclusion criteria were toxic-appearing patients, patients with apparent life- or limb-threatening illness or injury, patients meeting triage criteria for emergent care, patients with significantly abnormal triage vital signs (BPs less than 90/50 or greater than 200/100, heart rate less than 50 or greater than 100, oxygen saturation less than 94% or respiratory rate greater than 16), patients unable to give verbal consent, and patients unable to have cuff blood pressures done in either upper extremity.

The ultrasound screen and pressure monitor were video recorded and over-read by another investigator who was blinded to the initial URAC measurements.

### Description of Setup

**Figure 2:**
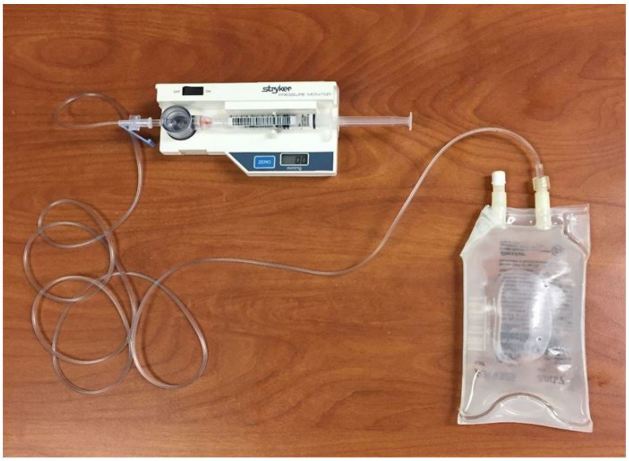
Noninvasive pressure measurement setup, showing 100mL bag of normal saline connected to Stryker pressure meter via standard intravenous (IV) tubing.

**Figure 3:**
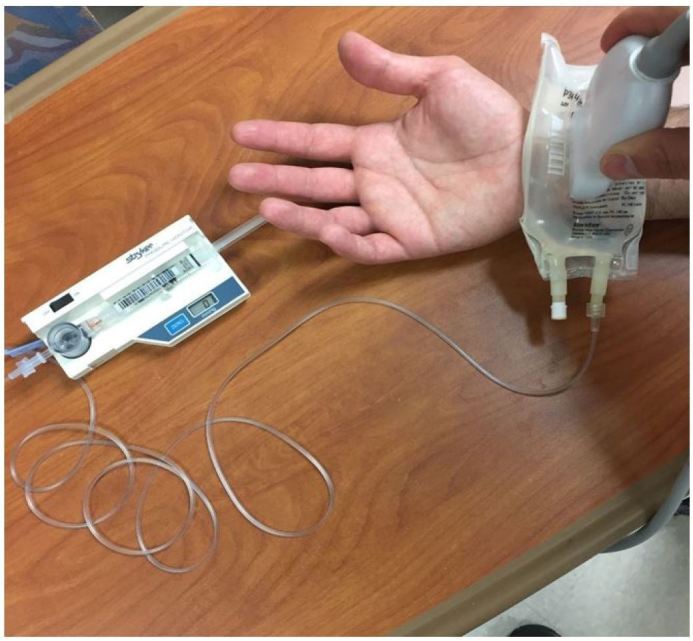
Radial artery is visualized through 100mL bag of normal saline with ultrasound probe and compression is applied to achieve coaptation and complete collapse of the artery wall

Patient was seated in a standard triage chair with an armrest. The radial artery was identified using a Zonare ZS-3 Ultrasound System (Mountain View, CA) and an L10-5 linear ultrasound transducer set to the vascular exam settings. A 100mL bag of normal saline was connected to a Stryker intra-compartmental pressure monitor using standard intravascular tubing and flushed with saline to remove any air (Figure 2). The 100mL bag was placed on the patient’s volar wrist overlying the radial artery (Figure 3). Ultrasound gel was applied between each layer. Pressure was slowly applied to the bag with the linear transducer, and the radial artery was observed for coaptation of the anterior and posterior walls and then complete collapse (Figure 4). We defined *coaptation* as the point at which the anterior and posterior walls of the pulsatile radial artery first touched and *complete collapse* as the point at which the radial artery no longer visibly opened or displayed pulsatility. The pressure reading on the Stryker monitor was recorded at the points of coaptation and complete collapse. The ultrasound screen and Stryker monitor were recorded with a Sony Handycam camcorder, which was reviewed by the principal investigator to independently identify the point of coaptation and complete collapse to assess agreement. The patient’s BP was then measured using a standard automated cuff. Patient level of comfort with the URAC method was also assessed, with patients reporting either “more,” “same” or “less” comfort compared to automated cuffs.

**Population Health Research Capsule**

What do we already know about this issue?

Venous pressure can successfully be assessed using compression ultrasound of peripheral veins. However, no studies have used compression ultrasound to assess arterial blood pressure

What was the research question?

Is ultrasound-guided radial artery compression (URAC) a feasible method to assess blood pressure?

What was the major finding of the study?

There is a statistically significant correlation between automated cuff and URAC diastolic blood pressure measurements.

How does this improve population health?

If found to be an acceptable method of assessing blood pressure, URAC could be a backup method to measure blood pressure and potentially replace invasive methods of assessment in select patients.

### Statistical Analysis

We performed data analysis using SPSS Statistics version 24.0 (IBM Corp. Released March 15 2016. IBM SPSS Statistics for Windows, Version 24.0. Armonk, NY). A paired samples t-test was performed for both systolic pressure (URAC vs cuff pressure) and diastolic pressure (URAC vs cuff pressure) to determine any statistically significant differences. We also compared the URAC and cuff pressures using the intraclass correlation coefficient. Interrater reliability between the two observers was calculated using the intraclass correlation coefficient.

## RESULTS

This study found a statistically significant difference between automated systolic pressure and URAC pressure for artery collapse (p = 0.02), but no statistically significant difference between automated diastolic pressure and URAC coaptation pressure (p = 0.46) (Table 1). Intraclass correlation was 0.48 (p=0.04) for systolic BP and 0.57 (p=0.02) for diastolic BP (Table 2). Of 25 patients sampled, 20 (80%) found the URAC method more comfortable than the cuff measurement (Table 3). The agreement between the two observers using intraclass correlation was 0.88 for identifying coaptation on ultrasound and 0.92 for identifying collapse (Table 4).

## DISCUSSION

We often guide the management of patients in the ED by their BPs. When unable to obtain accurate readings using automated methods, we spend precious time attempting different locations, using standard cuff manometry and occasionally resorting to invasive methods with its inherent risks. For these reasons a simple, non-invasive and reliable method of measuring BP is desirable for when the automated cuff fails.

As described by Thalhammer et al., compression sonography using special equipment is capable of accurately measuring venous pressure with peripheral veins.^4^ In addition, multiple studies have shown that the radial artery can be easily identified via ultrasound.^6^,^8^,^9^ We attempted to show that noninvasive peripheral arterial BP could be similarly assessed using common items found in most EDs.

This pilot study found a statistically significant moderate correlation between automated diastolic pressure and URAC measurement for coaptation pressure (p = 0.46). Furthermore, there was a high level agreement between two observers independently identifying the points of coaptation and collapse of the radial artery walls. The results show the URAC method has some promise as a reliable alternative method of BP assessment.

Given the focal pressure being applied to the arm with the URAC method, the secondary aim of this study was to compare patient’s comfort level during automated cuff and URAC measurements. Automated BP cuff measurements are commonly uncomfortable for the patient.^7^ The overwhelming majority of patients (80%) found the URAC method to be more comfortable than the automated cuff measurement, and none found it to be less comfortable.

The authors of this study did not undergo any special training for this procedure but are trained in emergency sonography with fellowship or equivalent training. However, previous studies have shown that vascular compression of the forearm can be successfully performed after brief training,^5^ and the authors feel the URAC technique could be easily mastered by novice sonographers.

## LIMITATIONS

This pilot study has several limitations. It consisted of a small convenience sampling of patients at a single urban ED, which limits its generalizability. As a standard, our study used automated cuff pressures instead of a more accurate invasive BP measurement. Patients with significantly abnormal BPs were excluded. The technique requires the observer to be comfortable with identification of the radial artery on ultrasound, image optimization, and maintaining an adequate image of the vein while applying pressure on the saline bag. For this pilot study we used tools commonly found in most EDs, but not all EDs may have a Stryker or other intra-compartmental pressure monitor available. The system would have to remain partially constructed to be efficient. However, if compression-ultrasound BP assessment is further validated, a simple and integrated device could be developed.

## CONCLUSION

Compression ultrasonography shows promise as a method for BP measurement. Further studies are needed and should target comparison of compression sonography measurements to more accurate standards. If proven reliable, compression ultrasonography could serve as a backup method for BP measurement, reduce the need for arterial line placement, and be integrated into workflows for efficient use.

## Figures and Tables

**Figure 1:**
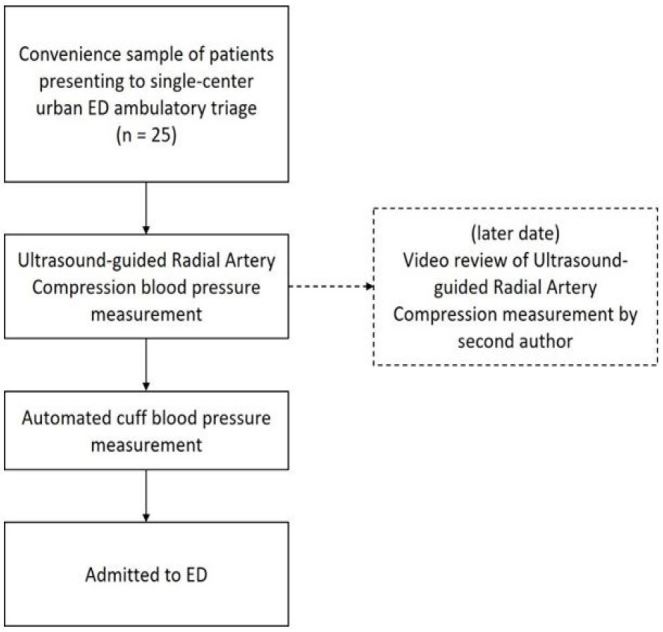
Patient flow diagram. *ED,* emergency department

**Figure 4a:**
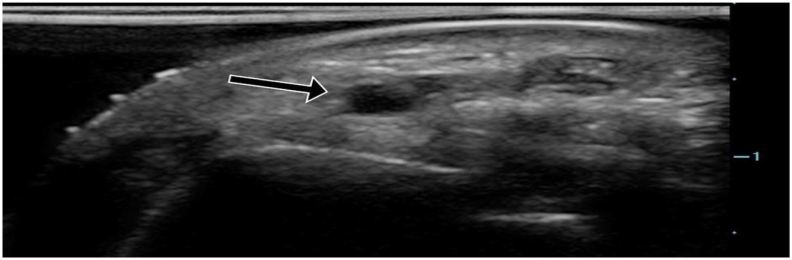
Ultrasound of the radial artery showing normal anatomy (arrow).

**Figure 4b:**
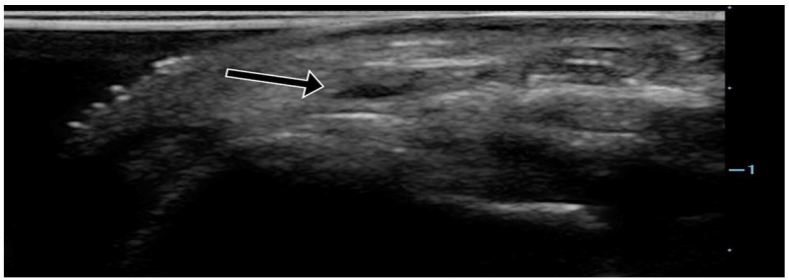
Ultrasound of the radial artery with pressure applied, beginning to show coaptation of the anterior and posterior walls representing diastolic pressure (arrow).

**Figure 4c:**
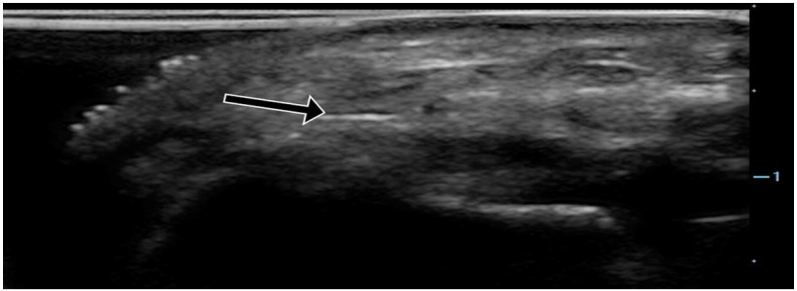
Ultrasound of the radial artery showing complete collapse with pressure applied, representing systolic pressure (arrow).

**Table 1:** Comparison of mean automated cuff pressure versus mean ultrasound-guided radial compression (URAC) pressure and corresponding p-values.

	Cuff pressure	URAC	p-value
Systolic	138.6 ± 22.1	126.9 ± 19.8	0.02
Diastolic	83.7 ± 13.0	86.5 ± 19.8	0.46

**Table 2:** Intraclass correlation between automated cuff and ultrasound-guided radial compression (URAC) systolic and diastolic blood pressure.

	Intraclass correlation	p-value
Systolic	0.48	0.04
Diastolic	0.57	0.02

**Table 3:** Automated cuff BP compared to ultrasound-guided radial compression (URAC) BP and comfort level.

	Cuff BP		URAC BP		Comfort
			
Patient	Systolic	Diastolic	Collapse	Coaptation	
1	162	106	160	122	More
2	145	80	165	125	More
3	139	79	114	79	Same
4	171	121	157	124	More
5	143	88	110	65	Same
6	130	90	111	72	More
7	145	91	132	100	More
8	130	82	153	116	Same
9	188	95	129	64	More
10	111	75	106	73	More
11	158	82	89	58	More
12	125	87	117	72	More
13	120	75	123	79	More
14	191	106	134	94	More
15	115	78	140	108	More
16	117	66	118	78	More
17	114	83	124	96	More
18	146	77	139	67	More
19	127	81	130	81	More
20	117	71	93	77	More
21	137	74	120	92	Same
22	152	88	152	91	Same
23	139	72	118	66	More
24	120	64	115	82	More
25	124	82	124	82	More

*BP*, blood pressure.

**Table 4:** Correlation between the two observers to identify on ultrasound the point of initial coaptation of radial arterial walls (diastole) and complete collapse (systole).

	Intraclass correlation
Coaptation (diastolic pressure)	0.88
Collapse (systolic pressure)	0.92
